# Coronavirus disease 2019 outbreak in Beijing’s Xinfadi Market, China: a modeling study to inform future resurgence response

**DOI:** 10.1186/s40249-021-00843-2

**Published:** 2021-05-07

**Authors:** Xiao-Li Wang, Xin Lin, Peng Yang, Zun-You Wu, Gang Li, Jennifer M. McGoogan, Zeng-Tao Jiao, Xin-Jun He, Si-Qi Li, Hong-Hao Shi, Jing-Yuan Wang, Sheng-Jie Lai, Chun Huang, Quan-Yi Wang

**Affiliations:** 1 Beijing Research Center for Preventive Medicine, Beijing Center for Disease Prevention and Control, Beijing, China; 2 School of Public Health, Capital Medical University, Beijing, China; 3 Beijing Municipal Key Laboratory of Clinical Epidemiology, Beijing, China; 4 School of Computer Science and Engineering, Beihang University, Beijing, China; 5 Chinese Center for Disease Control and Prevention, Beijing, China; 6 Yidu Cloud AI Lab, Yidu Cloud (Beijing) Technology Co., Ltd, Beijing, China; 7 Johns Hopkins Bloomberg School of Public Health, Johns Hopkins University, Baltimore, MD USA; 8 WorldPop, School of Geography and Environmental Science, University of Southampton, Southampton, UK; 9 School of Public Health, Key Laboratory of Public Health Safety, Ministry of Education, Fudan University, Shanghai, China

**Keywords:** Public health, Nonpharmaceutical intervention, COVID-19, SARS-CoV-2, Beijing

## Abstract

**Background:**

A local coronavirus disease 2019 (COVID-19) case confirmed on June 11, 2020 triggered an outbreak in Beijing, China after 56 consecutive days without a newly confirmed case. Non-pharmaceutical interventions (NPIs) were used to contain the source in Xinfadi (XFD) market. To rapidly control the outbreak, both traditional and newly introduced NPIs including large-scale management of high-risk populations and expanded severe acute respiratory syndrome coronavirus 2 (SARS-CoV-2) PCR-based screening in the general population were conducted in Beijing. We aimed to assess the effectiveness of the response to the COVID-19 outbreak in Beijing’s XFD market and inform future response efforts of resurgence across regions.

**Methods:**

A modified susceptible–exposed–infectious–recovered (SEIR) model was developed and applied to evaluate a range of different scenarios from the public health perspective. Two outcomes were measured: magnitude of transmission (i.e., number of cases in the outbreak) and endpoint of transmission (i.e., date of containment). The outcomes of scenario evaluations were presented relative to the reality case (i.e., 368 cases in 34 days) with 95% Confidence Interval (CI).

**Results:**

Our results indicated that a 3 to 14 day delay in the identification of XFD as the infection source and initiation of NPIs would have caused a 3 to 28-fold increase in total case number (31–77 day delay in containment). A failure to implement the quarantine scheme employed in the XFD outbreak for defined key population would have caused a fivefold greater number of cases (73 day delay in containment). Similarly, failure to implement the quarantine plan executed in the XFD outbreak for close contacts would have caused twofold greater transmission (44 day delay in containment). Finally, failure to implement expanded nucleic acid screening in the general population would have yielded 1.6-fold greater transmission and a 32 day delay to containment.

**Conclusions:**

This study informs new evidence that in form the selection of NPI to use as countermeasures in response to a COVID-19 outbreak and optimal timing of their implementation. The evidence provided by this study should inform responses to future outbreaks of COVID-19 and future infectious disease outbreak preparedness efforts in China and elsewhere.

**Graphical abstract:**

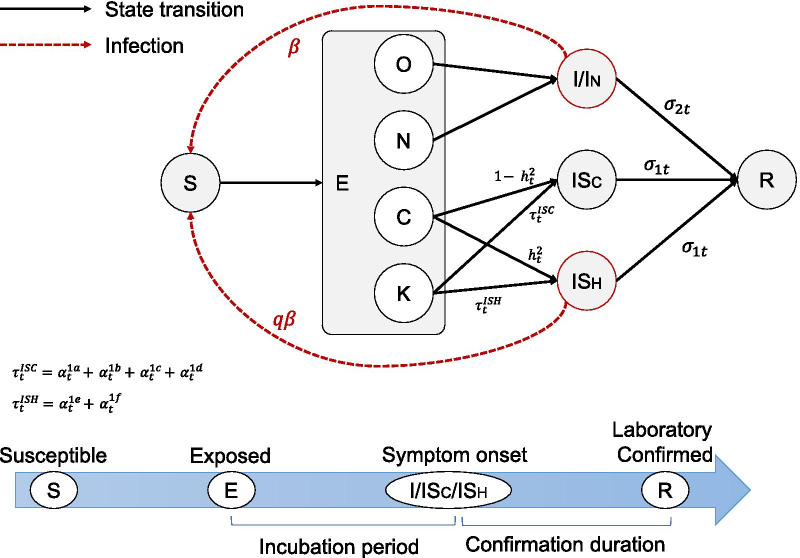

**Supplementary Information:**

The online version contains supplementary material available at 10.1186/s40249-021-00843-2.

## Background

Although it has been more than 12 months since the first confirmed case of novel coronavirus disease 2019 (COVID-19) was reported, and vaccines have been approved and rolled out in some countries to protect high-risk populations for severe outcomes, for the time being non-pharmaceutical interventions (NPIs) remain primary public health measures to slow the transmission and reduce the healthcare burden before vaccines are widely available and herd immunity can be achieved. Since the early stage of the pandemic, there have been a series of studies to understand the impacts of response policymaking and NPIs implementation on COVID-19, using mathematical modeling and simulations [1–3]. Different NPIs such as case isolation, close contact tracing and quarantine, social distancing, mask wearing, and travel restrictions have a varying effectiveness in controlling the transmission of COVID-19 across regions and time [4–10]. However, few studies investigated the resurgences of COVID-19 transmission and it is little known about the effectiveness of NPIs for controlling secondary waves in regions where the COVID-19 epidemic has been contained [11].

The COVID-19 outbreak in Beijing’s Xinfadi (XFD) Wholesale market occurred on June 11, 2020 after 56 consecutive days without a newly confirmed case in Beijing. The resurgence was brought under control in just 34 days from the onset of illness of the first known case (June 5, 2020) to zero new infections detected (July 10, 2020) [11]. It offers an exceptional opportunity to develop a model using real-world data and to quantitatively evaluate the timing and impact of integrated NPIs for containing COVID-19 resurgences. The source of this outbreak was identified as the virus spread from XFD market [12], the largest wholesale food market in Asia that has about 3000 workers and 50 000 visitors each day and provides about 80% of Beijing’s food supply. Within the massive XFD market complex, there are a total of 14 trading halls. One of which, the Beef and Mutton Trading Hall (BMTH), has been identified as the major infection source in this outbreak [12]. After the new outbreak was discovered on June 11, the municipal government have adopted a two-pronged approach—re-instating NPIs used during the initial wave in January–March 2020 and introducing new NPIs including: (i) large-scale tracing and management of high-risk populations identified by exposure risk levels, and (ii) expanded SARS-CoV-2 nucleic acid screening in the general population in Beijing.

The implementation of combining interventions have rapidly and successfully contained the resurgence and interrupted the transmission, and only 362 confirmed cases, 40 asymptomatic infections in Beijing and 34 linked infections outside Beijing were found, with zero deaths and less disruption to routine socioeconomic activities [11]. To assess the effectiveness of the response to the COVID-19 outbreak in Beijing’s XFD market and inform future response efforts of resurgence across regions, we developed a COVID-19 outbreak modeling framework to examine impacts of various identification timings and NPIs for this outbreak under hypothetical response scenarios.

## Methods

### Study design

We constructed a modified susceptible–exposed–infectious–recovered (SEIR) model to evaluate the effectiveness of NPIs in containing COVID-19 after the outbreak in Beijing’s XFD market. We specifically modeled four scenarios for two key outcomes: the magnitude of transmission and the endpoint of transmission. Our methods and results were reported according to guidelines developed by Bennett and Manuel [13]. Ethical approval and informed consent requirements were waived by the Institutional Review Board and Human Research Ethics Committee of the Beijing Center for Disease Prevention and Control (Beijing CDC) because this study was considered a continuation of the public health investigation associated with an emerging infectious disease.

### Data source

The details of the Beijing XFD market outbreak and NPIs implemented in response, have been previously described [11, 14]. The data used in this study were extracted from the Notifiable Infectious Disease Reporting System (basic individual case-level demographics, location, and diagnostic data), the Epidemiological Investigation Information System (detailed individual case-level exposure, symptom, and clinical data), and the Close Contacts Tracing and Management System (individual contact-level demographics, exposures, location, and quarantine data) [14]. A summary of Beijing XFD outbreak data used in developing the model is presented in Table 2.

The epidemiologic parameters such as incubation periods and contagious periods were calculated based on data from the epidemiological investigation and close contacts tracing and management. For incubation periods, we extracted 41 individual laboratory-confirmed records that have known dates of exposure. By combing the date of exposure with the date of symptom onset, we inferred the incubation periods for the 41 individual cases. We fitted the distribution of incubation periods to a Weibull distribution using a Maximum Likelihood Estimation method with R package *fitdistrplus* (https://www.jstatsoft.org/article/view/v064i04) [15]. The contagious periods were calculated as the average duration from symptom onset to laboratory confirmation, since once the infections were confirmed, they would be quarantined and not cause a secondary infection. These and other parameters and coefficients used for model simulation are based on the Notifiable Infectious Disease Reporting System and presented in Additional file 1: Table S1.

Key populations in this model were defined as the assessed stratified risk groups by exposure level in the XFD outbreak. XFD workers who were in the XFD market were assessed to be at the highest risk. They were traced through traditional epidemiologic investigation methods (i.e., face-to-face interviews or home visits) and quarantined in centralized facilities. Attack rate was calculated as the number of cases (numerator) divided by the number of total persons (denominator) presented as a percent. Attack rate among this high-risk group was 5.1%. The attack rate among workers in the BMTH was highest at 14.2%. Visitors to XFD market on June 12 were designated medium risk and quarantined in centralized facilities. They were found to have an attack rate of 0.1%. By contrast, visitors to XFD market before the outbreak (May 30–June 11) were assessed as low risk, traced by big data, and were asked to quarantine at home. The attack rate among this low-risk group was 0.02% (Table 1). Close contacts were defined as persons who had direct contact within one meter with a confirmed case four days before or any time after their symptom onset without personal protective equipment. The close contact population was excluded from the key population.

**Table 1:** Epidemiologic data collected for the XFD outbreak

Categories	Case numbers
Total infected cases during XFD outbreak	368
Key population	
Number of individuals in key population	546 000
Number of Infections identified from key population	272
Number of Infections identified via key population management	224
Close contacts	
Number of close contacts	6607
Number of close contacts with home-based quarantine	1055
Number of close contacts with centralized quarantine	5552
Number of infected close contacts	84
Number of infected cases identified via contact tracing	42
Expanded nucleic acid screening	
Number of people conducted nucleic acid screening	10 878 289
Number of Infected cases identified via expanded nucleic acid screening	28
Other methods	
Number of target population	10 111 711
Infected cases identified	76

### Model

**Fig. 1:**
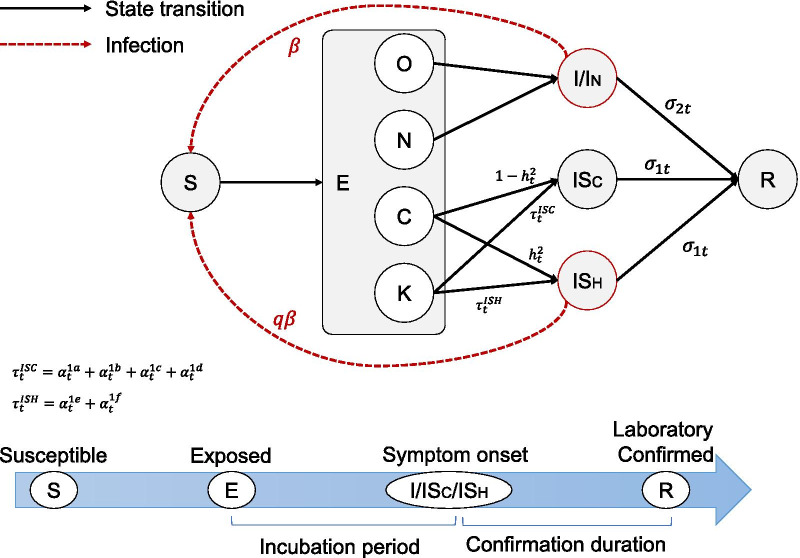
The illustration of the modified SEIR model. The model categorized the whole population into five subpopulations, susceptible (S), exposed and infected (not yet infectious) (E), infectious (I), infectious and isolated (IS) and removed (R). The IS group was further divided into two sub-populations according to the type of quarantine: those who were centralized quarantined (ISC) or home-based quarantined (ISH). E was further designated into a subpopulation K indicating key population to the XFD market, a subpopulation C indicating close contacts of confirmed cases, a subpopulation N representing the infections discovered by nucleic acid testing and a subpopulation O representing the infections identified by other methods

Our model categorized the whole population into five subpopulations, susceptible (*S*), exposed and infected (but not yet infectious; *E*), infectious (*I*), infectious and isolated (*IS*) and removed (*R*). Based on the actual situation in the XFD outbreak, we further divided *E* population into a subpopulation *K* indicating key population to the XFD market, a subpopulation *C* indicating close contacts of confirmed cases, a subpopulation *N* representing the infections detected by nucleic acid testing and a subpopulation *O* representing the infections identified by other methods. During this outbreak, a portion of the infected population was isolated early through close contact or key population tracing, or nucleic acid screening, so that it could not result in large scale secondary infection. Such infected population at the time of illness onset were classified as *IS* in our model. The *IS* group was further divided into two sub-populations according to the type of quarantine and became removed population R immediately: those who were quarantined in centralized facilities (*IS*_*C*_) and those who were in home-based quarantine (*IS*_*H*_). The removed population *R* additionally included the recovered (no deaths were reported during this outbreak). Under those assumptions, we developed a modified SEIR model illustrated in Fig. 1.

The equations are as follows:

1 $$\frac{d{S}_{t}}{dt}=-\beta {S}_{t}({I}_{t}+I{N}_{t})-q\beta {S}_{t}IS{H}_{t},$$

2 $$\frac{d{E}_{t}}{dt}=\beta {S}_{t}\left({I}_{t}+I{N}_{t}\right)+q\beta {S}_{t}IS{H}_{t}-\gamma {E}_{t}$$

3 $$\frac{d{I}_{t}}{dt}=\left(\gamma {E}_{t}-{K}_{t}-{C}_{t}-{N}_{t}\right)+\left(1-{\alpha }_{t}^{1a}-{\alpha }_{t}^{1b}-{\alpha }_{t}^{1c}-{\alpha }_{t}^{1d}-{\alpha }_{t}^{1e}-{\alpha }_{t}^{1f}\right){K}_{t}-{\sigma }_{2t}{I}_{t}$$

4 $$\frac{d{IN}_{t}}{dt}={N}_{t}-{\sigma }_{3t}{IN}_{t}$$

5 $$\frac{d{ISC}_{t}}{dt}=\left(1-{h}_{t}^{2}\right){C}_{t}+\left({\alpha }_{t}^{1a}+{\alpha }_{t}^{1b}+{\alpha }_{t}^{1c}+{\alpha }_{t}^{1d}\right){K}_{t}-{\sigma }_{1t}{ISC}_{t}$$

6 $$\frac{d{ISH}_{t}}{dt}={h}_{t}^{2}{C}_{t}+\left({\alpha }_{t}^{1e}+{\alpha }_{t}^{1f}\right){K}_{t}-{\sigma }_{1t}{ISH}_{t}$$

7 $$\frac{d{R}_{t}}{dt}={\sigma }_{2t}{I}_{t}+{\sigma }_{3t}{IN}_{t}+{\sigma }_{1t}\left({ISC}_{t}+IS{H}_{t}\right)$$

In the equation, *K*_*t*_ indicates the daily infections from the key population in XFD market, and ${\alpha }_{t}^{1a}, {\alpha }_{t}^{1b},{\alpha }_{t}^{1c},{\alpha }_{t}^{1d},{\alpha }_{t}^{1e} and {\alpha }_{t}^{1f}$ are the proportions of the daily infections in group 1.1 to group 3.2 (Table 2) of the total daily number in key population, respectively. ${C}_{t}= {\alpha }_{2}^{t}(\gamma {E}_{t}-{K}_{t})$ indicates the daily infections discovered by close contact tracing, while parameter ${\alpha }_{t}^{2}$ models the effect of the close contact tracing and management. It is the proportion of the daily infections of the daily total number from close contacts, excluding the cases from the key population of the XFD market. ${N}_{t}= {\alpha }_{3}^{t}(\gamma {E}_{t}-{K}_{t})$ indicates the daily infections found by expanded nucleic acid screening, while parameter ${\alpha }_{t}^{3}$ models the effect of the expanded nucleic acid screening. It is the infection proportion of daily total number from expanded nucleic acid screening, excluding the cases from the key population of the XFD market. Parameter ${\sigma }_{t}$ represents the speed of transition from the infectious to the removed. Since all the infectious cases/infections were isolated in designated hospitals or home once confirmed, ${\sigma }_{t}$ represents the speed of transition from the infectious to the confirmed. It equals the reciprocal of the time difference between the disease onset and confirmation. Parameter ${h}_{t}^{2}$ is the percentage of cases who were home-isolated among all the cases identified by closed contact tracing. These parameter values were abstracted from epidemiologic data (Tables 1, 2, Additional file 1: Table S1).

**Table 2:** Group of key population and attack rate of each group

Group	Exposed populations	Quarantine protocol	Tracing techniques	Risk level	Total no	No. of cases	Attack rate (%)
1	Workers at the XFD market	Centralized quarantine	Onsite	High	3311	169	5.10
1.1	Workers at the BMTH	Centralized quarantine	Onsite	High	838	119	14.20
1.2	Workers at the XFD market other than BMTH	Centralized quarantine	Onsite	High	2473	50	2.02
2	Visitors to the XFD market on June 12th	Centralized quarantine	Onsite	Medium	7689	8	0.10
2.1	Visitors to the BMTH market	Centralized quarantine	Onsite	Medium	1078	8	0.74
2.2	Visitors to the XFD market other than BMTH	Centralized quarantine	Onsite	Medium	6611	0	0
3	Visitors to XFD between May 30th and June 11th	Home quarantine	Big data	Low	535 000	95	0.02
3.1	Visitors to the BMTH market	Home quarantine	Big data	Low	75 000	64	0.09
3.2	Visitors to the XFD market other than BMTH	Home quarantine	Big data	Low	460 000	31	0.007

*BMTH* Beef and Mutton Trading Hall,* XFD* Xinfadi

Parameter $\beta$ is the transmission rate. Parameter $q$ denotes the reduction of infectiousness for home-quarantined patients compared to non-quarantined patients. We estimated $\beta$ and $q$ by Markov Chain Monte Carlo (MCMC) with the Adaptive Metropolis algorithm implemented in the Python package *PyMC* (version 2.3.8) [16, 17]. We used a non-informative flat prior of Uniform (9e-9, 5e-4) for $\beta$, and Uniform (0.01, 1.0) for $q$. We fitted the model with data of cumulative cases from June 6 to July 10, 2020, by the date of symptom onset. After a burn-in of 1,000 iterations, we ran the MCMC simulation for 30,000 iterations with a sampling size of each 10 iterations. The RMSE of cases from June 6 to July 10 between model prediction and observation is 16.61. All of these analyses were performed in Python (version 3.6.0 https://www.python.org/).

### Simulated scenarios

#### Timing of initiation comprehensive measures

We hypothesized that delays in the implementation of the NPI, infection source intervention, would result in higher magnitude of transmission and later endpoint of transmission. Therefore, we modeled a 3 day, 7 day, and 14 day delay or ahead of the timing of infection source identification. We compared these predicted results with the actual outcomes from the XFD outbreak response: 368-case transmission magnitude and July 10 transmission endpoint. We assumed that: (1) unlimited health resources, (2) all other NPIs implemented precisely as in the real outbreak response, and (3) the proportion of each group of key population in Table 2 remains unchanged. We also changed the timing of other NPIs accordingly in model simulation (timing of the closure of XFD market, quarantining of key population, close contact tracing, expanded nucleic acid screening).

#### Management of key population

There were 224 confirmed infections identified in all managed key population. We simulated the epidemic development under quarantine protocols to the market workers or visitors to the market, respectively. In our simulation, for simplicity, we assumed that other NPI measures were independently unchanged when evaluating the effectiveness of each NPI measure of interest.

#### Quarantine of traced close contacts

There were 42 confirmed infections identified among close contacts group. For close contacts tracing, we compared the effect of centralized quarantine versus home quarantine.

#### Expanded screening for the general population

There were 28 confirmed infections identified from expanded screening in the general population. Expanded nucleic acid screening was expanded from the center of the XFD outbreak to surroundings among the general population. We simulated the effect of timing of nucleic acid test with 3 or 7 days delayed or in advance.

For all scenarios, we repeated the simulations based on parameter values estimated by 30 000 MCMC iterations with sampling at each 10 steps (i.e., 3000 samples totally) to construct the 95% confidence intervals (95% *CI*) of the epidemic curve by the 2.5 and 97.5 percentiles at each time point. The simulation results were presented as mean values and 95% *CI* calculated from the 3000 MCMC samples in this study. All the analyses were conducted using Python software, version 3.6.0.

### Sharing

The data used in this study are from public accessible database, internal databases from Beijing CDC, as well as news briefings. The mathematic model and code used for the analysis are available by addressing to the corresponding authors.

## Results

### Timing of combination NPI implementation

**Fig. 2:**
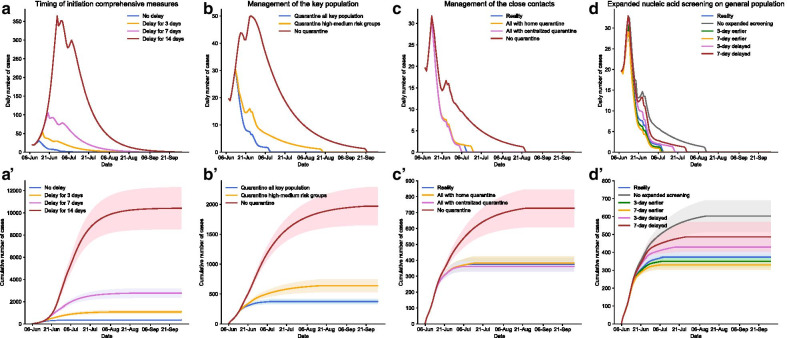
Scenario model simulation to assess the timing of intervention, management of key populations, contact tracing and expanded SARS CoV-2 Nucleic Acid screening. (**a**) and (**a’**) daily and cumulated total case number with the 3 day, 7 day, and 14 day delayed identification of XFD market as the source of infection. (**b**) and (**b’**) daily and cumulated total case number simulated for management on the key population to the XFD market. (**c**) and (**c’**) daily and cumulated total case number simulated for the close contacts traced with different quarantine protocols. (**d**) and (**d’**) daily and cumulated total case number simulated for delayed or earlier expanded Nucleic acid screening on general population. Shading areas indicate 95% confidence intervals

According to our model, a 3 day delay from the actual timing with which combination NPI response measures were initiated (i.e., identification of XFD as the infection source and initiation of NPIs) would lead to a threefold increase in the magnitude of transmission (95% *CI*: 2.6–3.4; i.e., 1104 vs 368 cases). A 7 day delay would lead to a 7.5-fold increase in the magnitude of transmission (95% *CI*: 6.4–8.6; 2768 vs 368 cases) and a 14 day delay a 28.2-fold increase (95% *CI*: 23.2–33.3; i.e., 10 411 vs 368 cases). Finally, these delays would also lead to endpoints of transmission delayed by 31, 50, and 77 days, respectively (Table 3, Fig. 2a and a′).

**Table 3:** Results of scenario simulations on two outcomes: magnitude of transmission and endpoint of transmission

	Magnitude of transmission				Endpoint of transmission
Scenarios simulated	Number of cases (95% *CI*)	Relative increase fold (95% *CI*)	Number of cases in target-population (95% *CI*)	Relative increase fold (95% *CI*)	Date of containment (Relative delay)
Timing of initiation comprehensive measures					
Reality: Delayed 0 days	368	-	368		July 10
Scenario 1: Delayed 3 days	1104 (947–1261)	3.0 (2.6–3.4)	1104 (947–1261)	3.0 (2.6–3.4)	August 10 (31 days)
Scenario 2: Delayed 7 days	2768 (2360–3176)	7.5 (6.4–8.6)	2768 (2360–3176)	7.5 (6.4–8.6)	August 29 (50 days)
Scenario 3: Delayed 14 days	10,411 (8549–12,272)	28.2 (23.2–33.3)	10 411 (8549–12 272)	28.2 (23.2–33.3)	September 25 (77 days)
Management of Key Population					
Reality:	368	-	224		July 10
Scenario 1: No quarantine	1,969 (1,658–2,280)	5.5 (4.5–6.2)	1825 (1514–2136)	8.2 (6.8–9.5)	September 21 (73 days)
Scenario 2: Quarantine high-medium risk	640 (536–744)	1.7 (1.5–2.0)	496 (392–600)	2.2 (1.8–2.7)	August 18 (39 days)
Quarantine of traced close contacts					
Reality: Centralized and home quarantine	368		42		July 10
Scenario 1: No quarantine	727 (609–844)	2.0 (1.7–2.3)	401 (283–518)	9.5 (6.7–12.3)	August 23 (44 days)
Scenario 2: Centralized quarantine for all	361 (330–392)	1.0 (0.9–1.1)	35 (4–66)	0.8 (0.1–1.6)	July 5 (-5 days)
Scenario 3: Home quarantine for all	382 (341–423)	1.0 (0.9–1.2)	56 (15–97)	1.3 (0.3–2.3)	July 14 (4 days)
Expanded nucleic acid screening in the general population					
Reality:	368		28		
Scenario 1: Accelerated 7 days	332 (305–358)	0.9 (0.8–1.0)	-8 (-35–18)	0	July 7 (-3 days)
Scenario 2: Accelerated 3 days	352 (319–384)	1.0 (0.9–1.0)	12 (-21–44)	0.4 (0–1.6)	July 8 (-2 days)
Scenario 3: Delayed 3 days	429 (377 to 481)	1.2 (1.0–1.3)	89 (37–141)	3.2 (1.3–5)	July 18 (8 days)
Scenario 4: Delayed 7 days	487 (405 to 568)	1.3 (1.1–1.5)	147 (65–228)	5.3 (2.3–8.1)	July 27 (17 days)
Scenario 5: No Nucleic Acid Screening	603 (516 to 690)	1.6 (1.4–1.8)	263 (176–350)	9.4 (6.3–12.5)	August 11 (32 days)

*CI* Confidence interval

### Management of key populations

Similarly, if no quarantine was instituted, the magnitude of transmission would be 5.5-fold greater (95% *CI*: 4.5–6.2; i.e., 1969 vs 368 cases) and the endpoint of transmission would be delayed 73 days compared to reality. If only the high- and medium-risk populations (i.e., Group 1 and Group 2) were quarantined, the magnitude of transmission would be 1.7-fold greater (95% *CI*:1.5–2.0; 640 versus 368 cases) and the endpoint of transmission would be delayed 39 days (Table 3, Fig. 2b and b′). If just the targeted key population is modeled, no quarantine would lead to 8.2-fold greater (95% *CI*: 6.8–9.5; i.e., 1825 vs 224 cases) magnitude of transmission and quarantine only for high- and medium-risk populations, a 2.2-fold greater (95% *CI*: 1.8–2.7; i.e., 496 vs 224 cases) magnitude of transmission (Table 3).

### Quarantine of traced close contacts

According to our model, no quarantine for close contacts would result in twofold greater (95% *CI*: 1.7–2.3; i.e., 727 vs 368 cases) magnitude of transmission and a 44 day delay in the endpoint of transmission. A more rigorous all-centralized quarantine for close contacts would result in 1.0-fold (95% *CI*: 0.9–1.1; i.e., 361 vs 368 cases) the magnitude of transmission and a 5 day acceleration in the endpoint of transmission. By contrast, a less rigorous all-home quarantine for close contacts would result in 1.0-fold greater (95% *CI*: 0.9–1.2; i.e., 382 vs 368 cases) magnitude of transmission and a 4 day delay in the endpoint of transmission (Table 3, and Fig. 2c and c′). If just the targeted key population is modeled, no quarantine for close contacts results in 9.5-fold greater (95% *CI*: 6.7–12.3; 401 vs 42 cases) magnitude of transmission. More rigorous all-centralized quarantine for close contacts would result in 0.8-fold (95% *CI*: 0.1–1.6; 35 vs 42 cases) the magnitude of transmission whereas less rigorous all-home quarantine would result in 1.3-fold (95% *CI:* 0.3–2.3; 56 vs 42 cases) the magnitude of transmission (Table 3).

### Expanded nucleic acid screening in the general population

According to our model, implementation of expanded nucleic acid screening 7 days earlier would result in 0.9-fold (95% *CI*: 0.8–1.0; 332 vs 368 cases) the magnitude of transmission and endpoint of transmission accelerated by three days. Three days earlier would result in 1.0-fold (95% *CI*: 0.9–1.0; 352 vs 368 cases) the magnitude of transmission and endpoint of transmission accelerated by two days. By contrast, a 3 day delay would result in 1.2-fold greater (95% *CI*: 1.0–1.3; 429 vs 368 cases) magnitude of transmission and an 8 day delay to the endpoint of transmission. A 7 day delay would result in 1.3-fold greater (95% *CI*: 1.1–1.5; 487 vs 368 cases) magnitude of transmission and a 17 day delay to the endpoint of transmission. Finally, no implementation of expanded nucleic acid screening at all would result in 1.6-fold greater (95% *CI*: 1.4–1.8; 603 vs 368 cases) magnitude of transmission and a 32 day delay to the endpoint of transmission (Table 3, and Fig. 2d and d′). If just the targeted key population is modeled, a 3 day acceleration yields 0.4-fold (95% *CI*: 0.0–1.6; 12 vs 28 cases) the magnitude of transmission whereas a 3 day delay yields 3.2-fold greater (95% *CI*: 1.3–5; 89 vs 28 cases) magnitude of transmission, a 7 day delay results in a 5.3-fold greater (95% *CI*: 2.3–8.1; 147 vs 28 cases) magnitude of transmission, and no implementation at all yields a 9.4-fold greater (95% *CI*: 6.3–12.5; 263 vs 28 cases) magnitude of transmission (Table 3).

## Discussion

Our results revealed that the delay in the identification of XFD as the infection source, subsequently leading a delay initiation of NPIs would have caused folds increase in transmission and months delay in the resurgence containment. A failure to implement the quarantine scheme employed in the XFD outbreak for at-risk groups would have caused greater transmission and more than two months of delay to containment. Similarly, failure to implement the quarantine plan executed in the XFD outbreak for close contacts would have caused greater transmission and a more than one month delay to containment. Finally, failure to implement expanded nucleic acid screening would have yielded greater transmission and about one month delay to containment. The evidence should inform suggestions to future resurgence outbreaks of COVID-19 and infectious disease outbreak preparedness efforts in China and worldwide.

Among the 272 infections found among the ~ 546 thousand individuals in defined key populations, 224 (82.4%) were identified through key population management while just 48 (17.6%) were identified through healthcare seeking, contact tracing, expanded nucleic acid screening, or other methods. Our results show that failure to manage these key populations would have caused an eightfold increase in number of cases and a 2 month delay to containment, indicating that this NPI is crucial. It is furthermore important to note that this kind of intervention requires no sample collection, no laboratory testing, and no detailed epidemiological investigation. Rather, it is practical, straightforward, and highly targeted.

More recently, the cities of Wuhan and Qingdao in China have undertaken population-wide nucleic acid screening for SARS-CoV-2 among all residents in the cities, but this kind of action is costly and thus controversial [18, 19]. To be practical, real-time risk assessments must be paired with expanded nucleic acid screening so that efficiency is optimized. To control costs yet identify infections with maximum efficiency, the authorities in Beijing applied nucleic acid screening with real-time adjustment based on risk levels, expanding screening from the epicenter, XFD market, to surroundings gradually and ending it when the daily positivity rate fell to zero. Although our results seemed to indicate only a small 1.6-fold increase in transmission with failure to implement expanded nucleic acid screening, a 9.4-fold increase was predicted by our model for the key populations if no nucleic acid screening was conducted in the general population. Considering improved efficiency of expanded screening when coupled with ongoing risk assessment, this NPI should also be considered important for achieving containment of a COVID-19 outbreak.

Management of close contacts, whether in home or centralized quarantine, is also a controversial topic. Yet, close contact management has been proven to be effective [20]. While home quarantine is more likely to cause secondary cases than centralized quarantine [20], centralized quarantine is much costlier. Moreover, centralized quarantine might trigger infection cluster, or even outbreak if the prevention and control measures are not effectively performed. Considering the cost of quarantine and the quality of life for quarantined persons, home-quarantine could be recommended if it could be conducted strictly.

This study has some limitations. In our simulations, we assumed that resources were adequate and all the NPIs were effectively implemented even as the numbers of cases surged. In reality, as disease transmission increased, some resources (e.g., centralized quarantine facilities) would be depleted and eventually reach a shortage. In addition, for simplicity, we assessed one NPI at a time, assuming the others would not be impacted. However, in reality, there are interdependencies between NPIs and the ability for officials to manipulate just one NPI alone at a time is limited. We also only evaluated two outcomes (i.e., magnitude and endpoint of transmission). Although these two are important with respect to containment, they fail to take other factors into account and ignore other outcomes of interest. Further study should be conducted using this model to evaluate the NPIs in different ways. For example, expanded nucleic acid screening in the general population appeared in our results to have only a small impact on the magnitude and endpoint of transmission. However, the extremely low positivity rate among the general population helped accelerate re-normalization and return to routine economic activity and social life in Beijing, limiting the negative consequences of anti-COVID-19 response activities. This socioeconomic benefit should be considered when evaluating NPIs in the future. Finally, social and culture factors also contributed to the success of containment efforts in the XFD market outbreak. Even though COVID-19 in China was at low level of transmission, and no new infections had been found in Beijing for nearly two months, residents were still wearing masks, obeying social distancing recommendations, and taking other personal prevention measures. They were also aware of the danger of SARS-CoV-2 infection. Thus, implementation of NPIs in the XFD outbreak was relatively well accepted, which may differ from circumstances in other settings in which this model may be applied.

## Conclusions

This modeling study provides important new evidence that can not only in form the selection of NPI to use as countermeasures in response to a COVID-19 outbreak but also can inform the optimal timing of their implementation. Moreover, this study clearly calculates the consequences of inaction and hesitation on the part of outbreak response teams and decision-making officials. This evidence should inform responses to future outbreaks of COVID-19 and future infectious disease outbreak preparedness efforts in China. Additionally, these lessons and this methodology can be used by other nations as they work to improve their anti-COVID-19 strategies and tactics.

## Supplementary Information

**Additional file 1: Table S1**. Parameters and coefficients for model simulation.

## Data Availability

The data used in this study were extracted from the Notifiable Infectious Disease Reporting System (basic individual case-level demographics, location, and diagnostic data), the Epidemiological Investigation Information System (detailed individual case-level exposure, symptom, and clinical data), and the Close Contacts Tracing and Management System (individual contact-level demographics, exposures, location, and quarantine data) [15].

## Abbreviations

Beijing CDC: Beijing Center for Disease Prevention and Control
BMTH: Beef and Mutton Trading Hall
*CI*: Confidence interval
COVID-19: Coronavirus disease 2019
MCMC: Markov Chain Monte Carlo
NPI: Non-pharmaceutical intervention
RMSE: Root-mean-square error
SARS-CoV-2: Severe acute respiratory syndrome coronavirus 2
SEIR: Susceptible–exposed–infectious–recovered
XFD: Xinfadi
