# Supplementary material for: Coronavirus disease 2019 outbreak in Beijing’s Xinfadi Market, China: a modeling study to inform future resurgence response
Source: Infect Dis Poverty. 2021 May 7;10:62. doi: 10.1186/s40249-021-00843-2 (PMC8103671; doi:10.1186/s40249-021-00843-2)
Supplement: Supplementary file 1 — Additional file 1: Table S1. Parameters and coefficients for model simulation. [file 40249_2021_843_MOESM1_ESM.docx]

Additional data

**Table S1**. Parameters and coefficients for model simulation

|  | Description | Value | Data Source |
| --- | --- | --- | --- |
| 1 | Start date of model simulation ($t_{0}$) | 2020/6/6 | Beijing CDC |
| 2 | Number of Susceptible (S) individuals at the initial day of simulation | 22000000 | Beijing Municipal Bureau of Statistics |
| 3 | Number of exposed (E) individuals at the initial day of simulation | 78 | Calculated from XFD COVID-19 records of onset date with incubation period distribution (Parameters 7, 8) |
| 4 | Number of infectious (I) individuals at the initial day of simulation | 5 | Calculated from XFD COVID-19 records of onset date |
| 5 | Number of removed (R) individuals at the initial day of simulation | 0 | Calculated from XFD COVID-19 records of confirmation date |
| 6 | Number of symptomatic key population at the initial day of simulation (K) | 2 | Calculated from XFD COVID-19 records of types of cases |
| 7 | Alpha of Weibull distribution for incubation period (γ) | 1.8175 | Distribution Fitted from XFD COVID-19 records of exposed date and onset date |
| 8 | Beta of Weibull distribution for incubation period (γ) | 6.2301 | Distribution Fitted from XFD COVID-19 records of exposed date and onset date |
| 9 | Average contagious periods (days) for home-quarantined patients ($\sigma_{1t}$) | 3.89 | Calculated from XFD COVID-19 records of onset, confirmation date and types of cases |
| 10 | Average contagious periods (days) for unquarantined patients ($\sigma_{2t}$) | 3.64 | Calculated from XFD COVID-19 records of onset, confirmation date and types of cases |
| 11 | Average contagious periods (days) for patients from expanded nucleic acid screening ($\sigma_{3t}$) | 4.60 | Calculated from XFD COVID-19 records of onset, confirmation date and types of cases |
| 12 | Ratio of patients discover by close contact tracing among all patients exclude key population ($\alpha_{t}^{2}$) | 0.2838 | Calculated from XFD COVID-19 records of types of cases |
| 13 | Ratio of patients discover by expanded nucleic acid screening among all patients exclude key population ($\alpha_{t}^{3}$) | 0.1892 | Calculated from XFD COVID-19 records of types of cases |
| 14 | Ratio of home-quarantined patients among all patients discovered by close contact tracing ($h_{t}^{2}$) | 0.8571 | Calculated from XFD COVID-19 records of types of cases and quarantine |
| 15 | Initialization of transmission rate ($\beta_{0}$) | 2.05E-08 | Trial-and-error |
| 16 | Initialization of infectivity reduction for home-quarantined patients ($q$) | 0.18 | Trial-and-error |
